# Use of Trichloroacetic Acid in Treatment of Atrophic Acne Scars: A Scoping Review

**DOI:** 10.1111/jocd.71110

**Published:** 2026-08-04

**Authors:** Mariana Garcia da Ponte Melo, Joyce Magalhães de Barros, Ramille Araújo Lima

**Affiliations:** ^1^ Postgraduate Program in Dental Sciences Christus University Fortaleza Ceará Brazil; ^2^ Postgraduate Program in Dentistry (PPGO) Federal University of Ceará (UFC) Fortaleza Brazil

**Keywords:** acne scars, chemical peeling, CROSS technique, scoping review, trichloroacetic acid

## Abstract

**Background:**

Atrophic acne scars represent a significant aesthetic and therapeutic challenge due to their multifactorial nature and morphological diversity. Trichloroacetic Acid (TCA) stands out as a versatile option, either through chemical peels or the Chemical Reconstruction of Skin Scars (CROSS) technique.

**Aim:**

To map scientific evidence regarding clinical protocols, concentrations, and efficacy of TCA, alone or in combined therapies, for atrophic acne scars.

**Methods:**

This scoping review followed the Joanna Briggs Institute (JBI) methodology and PRISMA‐ScR guidelines. Search strategies were applied across nine databases using the PCC strategy. Thirty‐four studies published between 2002 and 2025 were included.

**Results:**

The CROSS technique was the most evaluated protocol (58.8% of studies), with concentrations ranging from 50% to 100%; it demonstrated high selectivity and efficacy, particularly for ice‐pick and deep boxcar scars. Chemical peels accounted for 41.2% of studies, with superficial concentrations (10%–20%) used for texture improvement and medium concentrations (25%–50%) for shallow atrophic lesions. Combined therapies (52.9%) highlighted the synergy between TCA (15%–20%), microneedling, and subcision. Reported adverse events were primarily transient, including post‐inflammatory hyperpigmentation (PIH) and prolonged erythema.

**Conclusion:**

TCA is a versatile, cost‐effective, and evidence‐backed tool. The findings highlight a definitive shift toward personalized multimodal approaches, where the CROSS‐technique's precision and the synergistic effects of combined therapies optimize dermal remodeling and patient outcomes.

## 1 Introduction

Acne vulgaris is a chronic inflammatory condition whose most persistent sequelae comprise atrophic scars, resulting from inadequate collagen synthesis during healing process [1, 2]. The clinical management of these scars represents a relevant therapeutic challenge [3, 4] and generally involves minimally invasive approaches, such as microneedling, subcision, lasers, dermal threads, and chemical peels, with search for effective and safe protocols being a central objective in clinical practice [5, 6].

In this context, trichloroacetic acid (TCA) stands out as a versatile agent capable of inducing neocollagenesis and dermal thickening, whether applied alone or through the CROSS (Chemical Reconstruction of Skin Scars) technique [7, 8]. Considering its multifactorial nature, as well as clinical manifestations of different depths and morphologies that rarely respond to a single intervention modality [5, 9, 10, 11, 12, 13], there is an increasing use of TCA for atrophic acne scars aiming to enhance tissue remodeling and optimize clinical results [9, 14], primarily in combined protocols that explore distinct mechanisms of action [3, 12, 15, 16].

Despite its wide clinical use, there is still significant heterogeneity in protocols, including variations in TCA concentrations and application regimens, as well as distinct therapeutic combinations [1, 5, 17]. Therefore, this scoping review aims to systematically map the evidence regarding use of TCA in atrophic acne scars, identifying the most used clinical parameters and knowledge gaps to support development of standardized and evidence‐based protocols.

## 2 Material and Methods

This scoping review was conducted according to methodology proposed by Joanna Briggs Institute and reported following PRISMA‐ScR guidelines. The research question was: “What are the clinical protocols and available evidence regarding the use of TCA, alone or in combined therapies, for treatment of atrophic acne scars?”

A search was performed in PubMed/MEDLINE, Embase, Scopus, Web of Science, LILACS, SciELO, Cochrane Library, ClinicalTrials.gov, and Google Scholar databases, without restrictions of language or year of publication, with search strategy adapted according to each database: (“Acne Scars” OR “Atrophic Acne Scarring”) AND (“Trichloroacetic Acid” OR “TCA” OR “TCA CROSS” OR “Chemical Reconstruction of Skin Scars”).

Were included randomized and non‐randomized clinical trials, comparative studies, cohort studies, and case series, as well as laboratory or experimental studies that evaluated TCA at different concentrations and application techniques, alone or in association with other therapies. Reviews, studies that evaluated other treatment modalities, duplicate records, or studies with insufficient methodological information were excluded.

**FIGURE 1:**
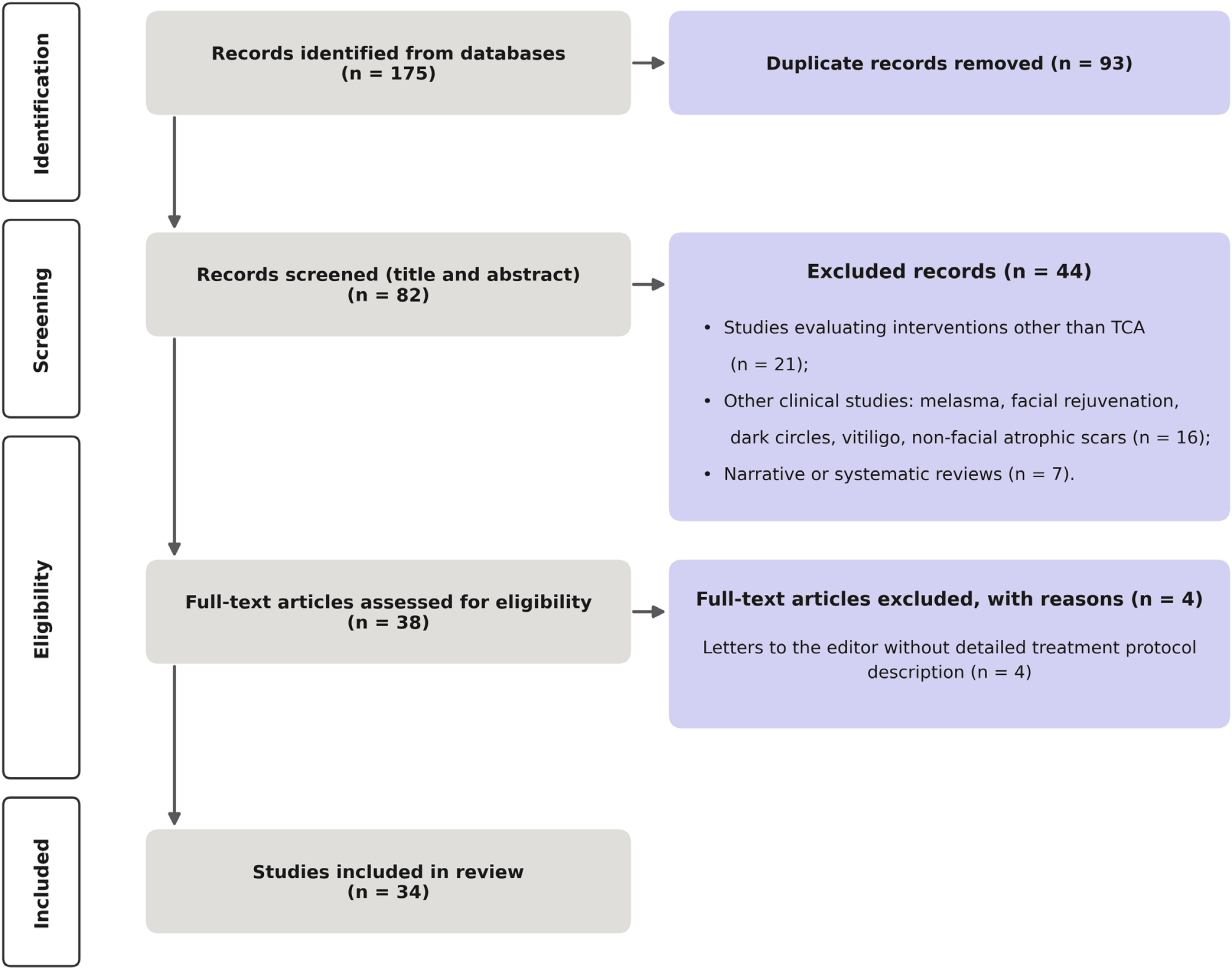
PRISMA‐ScR flow diagram of study selection. The diagram illustrates the identification, screening, eligibility, and inclusion phases of this scoping review according to PRISMA‐ScR guidelines. From 175 initial records, 93 duplicates were removed and 82 screened by title and abstract. After assessing 38 full‐text articles, four letters to the editor were excluded, resulting in a total of 34 included studies. *Source:* Authors' own illustration.

Study selection and data extraction were performed independently by two reviewers and 34 were incorporated into this review (Figure 1; Table 1).

**TABLE 1:** Characteristics of studies evaluating trichloroacetic acid‐based protocols for treatment of atrophic acne scars.

Author (year)	Country	Study type	*n*	Protocol/intervention	TCA concentration	Main findings	Adverse events
Soysal (2025)	Turkey	Clinical trial	40	CROSS (70% vs. 100%)	70%, 100%	100% more effective; 70% better tolerated	Intense burning; prolonged frosting (100%)
Manjhi (2024)	India	Clinical study	30	TCA vs. glycolic peel	30%	TCA deeper and more effective for remodeling	Intense peeling
Roy (2024)	India	Comparative	60	CROSS (TCA vs. glycolic acid)	65%	TCA 65% superior to glycolic 50%	Transient PIH; mild erythema
Solanki (2023)	India	RCT	30	MN + TCA peel	15%	Safe in high phototypes; 3 sessions needed	Mild erythema; fine peeling
Al‐Hamamy (2023)	Iraq	Clinical study	10	CROSS + MN	100%	High efficacy in grade III/IV scars	Mild PIH
Jangir (2023)	India	Prospective	50	Chemical peel	20%	Consistent atrophic scar reduction	PIH in 12% (resolved)
Ghassemi (2022)	Iran	RCT	45	CROSS + topical timolol	100%	Timolol reduced duration/severity of PIH	PIH (47%–80% across groups)
Dayal (2022)	India	RCT	40	MN + TCA peel	15%	Comparable efficacy to glycolic acid (GA showed added texture benefit)	Mild erythema; edema
Horovitz (2022)	Israel	Case series	6	Painting CROSS	85%	26.3% average volumetric reduction in scars	Mild erythema; PIH
Pakla‐Misiur (2021)	Poland	RCT	120	MN + peel (PRX‐T33)	Combined	Combination superior to isolated methods	No major adverse effects reported
Zayed (2021)	Egypt	Comparative	20	Subcision + peel/MN	35%	Effective in boxcar and rolling scars	Pain and edema (subcision‐related)
Al‐Hamamy (2021)	Iraq	Clinical study	13	Peel + dermasanding	25%	Safe for superficial remodeling	Persistent erythema (*n* = 2)
Mumtaz (2021)	Pakistan	Controlled trial	92	CROSS vs. PRP	50%	PRP significantly superior to CROSS	Not reported (safety not assessed)
Bahl (2020)	USA	Retrospective	25	CROSS + NAFL	70%–100%	Moderate to excellent improvement in majority of patients	None permanent
Abdel‐Magiud (2020)	Egypt	Clinical study	15 (TCA arm; 70 total study)	CROSS + biochemistry	80%	Decreased serum collagen III (correlates with clinical improvement)	Crusts; transient PIH
El‐Domyati (2018)	Egypt	Split‐face RCT	24	MN + TCA	15%	Histology: higher new collagen content	Mild erythema; edema
Agarwal (2015)	India	Clinical study	53	Focal CROSS	70%	Significant clinical improvement	Transient PIH in 15%
Dalpizzol (2016)	Brazil	Comparative	16	TCA vs. phenolic acid	90%	Both effective; phenol had fewer severe complications than TCA	Mild edema; PIH (*n* = 1)
Puri (2015)	India	RCT	50	Jessner + TCA vs. TCA	20%	Greater but not statistically significant improvement vs. TCA alone	Intense stinging; deep peeling
Garg (2014)	India	Original study	50	Subcision + MN + TCA	15%	Success in grade 2; improv. in 3–4 sessions	Erythema, edema (post‐dermaroller); PIH in 6%
Ahmed (2014)	Egypt	RCT	28	CROSS vs. CO_2_ laser	100%	CO_2_ laser significantly more effective than CROSS	Itching; infection (6/14); PIH (64%)
Leheta (2014)	Egypt	RCT	39	MN + TCA vs. 1540 nm	20%	78.3% reduction with combined laser+PCI + TCA (vs. 59.8%–61.8% for individual modalities)	Transient erythema
Leheta (2014)	Egypt	RCT	24	MN + TCA vs. phenol	20%	Similar efficacy to phenol; safer profile	Mild stinging; erythema
Aamir (2013)	Pakistan	Clinical study	9	CROSS monotherapy	35%	Safe, effective, low‐cost for rolled/atrophic scars	Prolonged erythema (*n* = 3)
Garem (2013)	Egypt	Prospective	30	CROSS	50%	Significant improvement (ice‐pick/boxcar)	Mild erythema; transient PIH
Khunger (2011)	India	Prospective	30	CROSS (Phototypes IV‐V)	100%	> 70% improvement; priming is essential	PIH in 2 patients
Leheta (2011)	Egypt	RCT	30	PCI vs. CROSS TCA	100%	Both protocols showed high efficacy	Transient erythema; crusts
Kang (2009)	S. Korea	Pilot study	10	Triple therapy^a^	100%	All patients showed clinical improvement	None significant
Kim (2009)	S. Korea	Split‐face RCT	20	CROSS vs. Er:Glass laser	100%	Laser superior for rolling scars; comparable to CROSS for ice‐pick scars	Prolonged erythema (CROSS side)
Fabbrocini (2008)	Italy	Clinical study	5	CROSS for ice‐pick	50%	50% sufficient for Grade 3 scars	None reported
Cho (2006)	S. Korea	Experimental	5	Histometric (Animal)	100%	Significant fibroblast activation	Expected crusts/edema
Yug (2006)	USA	Histological	3	Histological CROSS	95%	Increased Collagen I; fragmentation of elastic fibers	None relevant
Carniol (2005)	USA	Prospective	9	Laser + TCA peel	30%	TCA enhanced laser resurfacing effect	None reported
Lee (2002)	S. Korea	Cohort study	65	Original CROSS study	65%–100%	Established dose‐dependent efficacy	Erythema; transient PIH

Abbreviations: CROSS, chemical reconstruction of skin scars; MN, microneedling; NAFL, non‐ablative fractional laser; PIH, post‐inflammatory hyperpigmentation; PRP, platelet‐rich plasma; RCT, randomized controlled trial; TCA, trichloroacetic acid.

^a^ Triple therapy: dot peeling (CROSS), subcision, and fractional laser.

## 3 Results

This scoping review identified 34 studies (2002–2025) on TCA‐based treatments for atrophic acne scars, predominantly from Asia and the Middle East, comprising diverse study designs, including randomized clinical trials, comparative studies, and case series (Table 1).

### 3.1 TCA Monotherapy and CROSS Technique

**FIGURE 2:**
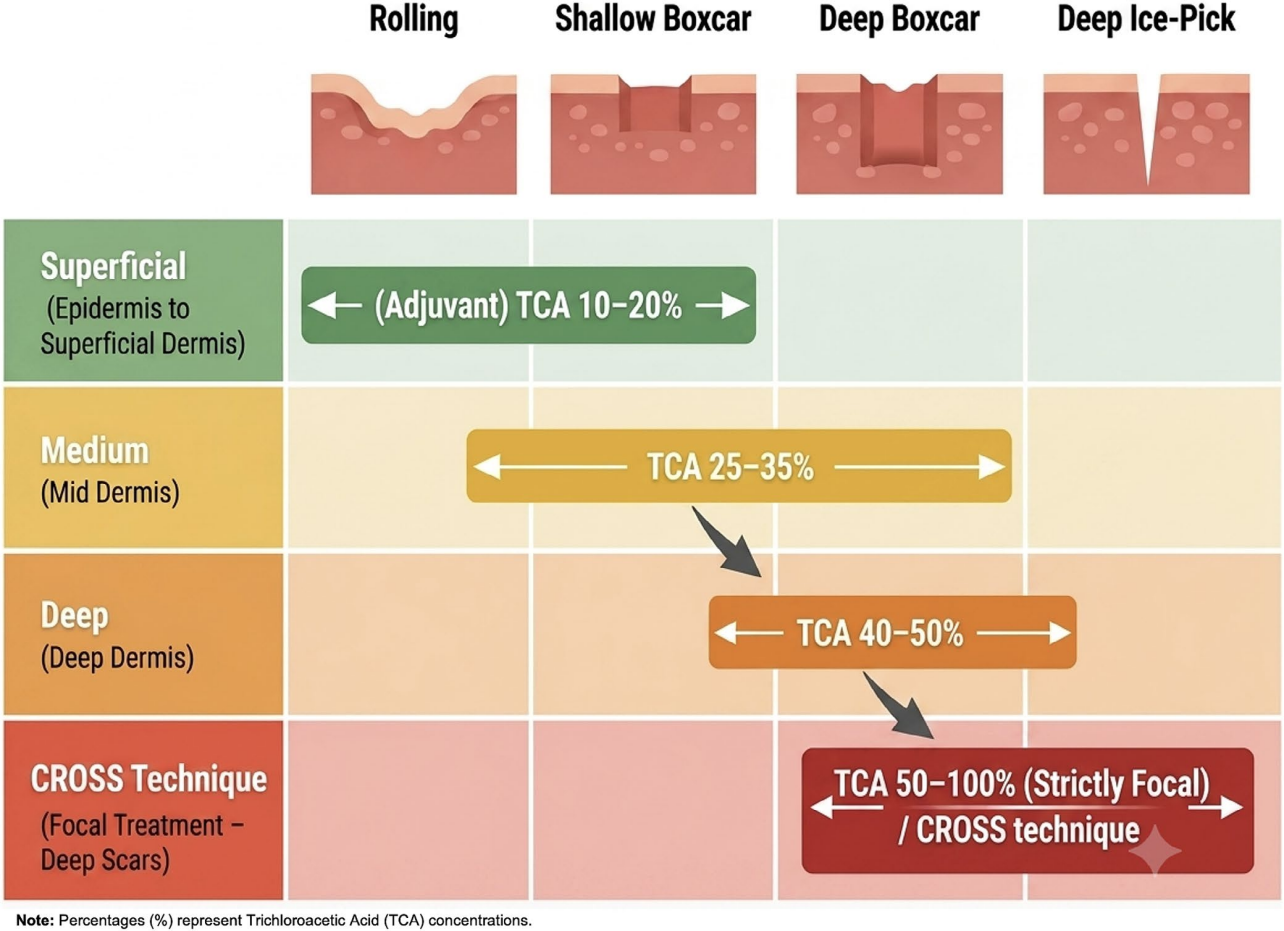
Schematic representation of atrophic acne scar morphologies and corresponding trichloroacetic acid (TCA) therapeutic protocols. This schematic diagram provides a practical algorithm for selecting appropriate TCA‐based interventions based on scar morphology, anatomical depth, and technical approach. Horizontal arrows indicate the TCA concentrations most used for each subtype. While lower concentrations (10%–35% superficial‐to‐medium peels) are shown alongside rolling and shallow boxcar scars, their use in rolling scars is typically recommended as an adjuvant tool within combined therapy protocols rather than monotherapy. Deeper boxcar scars may benefit from 40% to 50% deep peels, whereas deep ice‐pick scars strictly require focal, high‐concentration applications (50%–100%) via the CROSS technique to avoid widespread dermal damage. *Source:* Authors' own illustration.

Fourteen studies (41.2%) evaluated TCA as a single chemical peel, employing different concentrations according to scar type and desired depth [5, 11, 12, 13, 18, 19]. Protocols ranged from 10% and 20% [5, 11] to superficial peels and 25% to 50% to medium and deep peels [19, 20], showing efficacy for improving skin texture, superficial irregularities, residual hyperpigmentation, and shallow scars (Figure 2; Table 2). Use of 15% TCA combined with microneedling resulted in improvement in skin texture, with efficacy comparable to glycolic acid peeling combined with microneedling [5] and 20% TCA alone demonstrated promoting gradual clinical improvement of superficial scars [11]. Medium peels (35%–50%) were mainly described in older studies, with more efficacy in moderately deep scars [19].

**TABLE 2:** Classification of TCA therapeutic modalities according to depth and technique.

Technique	Histological depth	TCA concentration	Clinical indications
Superficial peel	Stratum corneum to granular layer	10%–20%	Superficial scars, rolling scars, texture irregularities
Medium peel	Full epidermis to papillary dermis	25%–35%	Shallow atrophic scars, superficial boxcar, and ice‐pick scars
Deep peel	Papillary to superficial reticular dermis	40%–50%	Moderately deep scars, deep boxcar scars
CROSS technique	Papillary to mid‐reticular dermis^a^	50%–100% (focal)	Deep ice‐pick scars; focal atrophic remodeling

*Note:* Depth of action refers to the expected histological response described in the included studies.

Abbreviations: CROSS, chemical reconstruction of skin scars; TCA, trichloroacetic acid.

^a^ The depth in CROSS is focal and depends on the pressure and volume of the acid applied within the scar unit.

CROSS technique was investigated in 20 studies (58.8%), corresponding to the most frequently evaluated protocol, especially in ice‐pick and boxcar scars [7, 18, 19, 20, 21, 22, 23, 24] (Table 2). CROSS, with TCA concentrations from 50% to 100%, demonstrated dose‐dependent dermal remodeling, offering advantages in cost and accessibility compared with ablative laser technologies [1, 7, 19]. Although the 100% concentration was associated with a slightly superior clinical response, 70% TCA presented shorter frosting time, less intraoperative discomfort, and better overall tolerability [1]. A case series conducted in Israel described an interesting technical modification called “painting CROSS”, with measurable volumetric reduction through three‐dimensional (3D) analysis and, consequently, greater control of scar edges [14].

### 3.2 Combined Therapies

Multimodal approaches (52.9% of protocols) were a consistent trend for complex scar morphologies [3, 5, 9, 10, 12] (Table 3). Combinations of TCA with microneedling (MN) and subcision being used predominantly in boxcar and rolling scars [3, 5, 9, 12]. The combination of TCA + MN promoted greater neocollagenesis and superior clinical improvement when compared to MN alone [9, 12]. The combination of TCA with subcision was associated with a higher incidence of transient pain and edema; however, these adverse effects were attributed to the mechanical nature of the procedure rather than the chemical action of the acid itself [25]. Furthermore, multimodal protocols integrating TCA, subcision, and energy‐based devices demonstrated superior outcomes for complex and mixed scars when compared to monotherapies [15, 26].

**TABLE 3:** Modalities of combined therapies with trichloroacetic acid for the treatment of atrophic acne scars.

Combined therapy	Studies/total (%)	Scar types	Synergy rationale	Main clinical findings
TCA + Microneedling (MN)	9/34 (26.5%)	Ice‐pick, boxcar, rolling	Microchannels enhance TCA transepidermal permeation	More homogeneous improvement; increased neocollagenesis
TCA + Subcision	4/34 (11.8%)	Boxcar, rolling	Mechanical release of fibrous bands + chemical remodeling	Reduction in scar depth; improved texture in deep lesions
TCA + Subcision + MN	2/34 (5.9%)	Mixed scars	Trimodal approach: mechanical, microperforation, and chemical	Consistent improvement in Grade II–IV scars
TCA + Energy‐based devices	2/34 (5.9%)	Mixed scars	Combined thermal and chemical stimuli	TCA acts as a complementary dermal modulator to laser/RF
TCA + Other chemical agents	1/34 (2.9%)	Mixed scars	Keratolysis (e.g., Jessner's) for uniform acid penetration	Deeper penetration; superior efficacy to TCA monotherapy

*Note:* Percentages refer to the proportion of studies included in this scoping review (*n* = 34). Energy‐based devices include fractional lasers and CO_2_ pinpoint irradiation.

Abbreviations: MN, microneedling; TCA, trichloroacetic acid.

### 3.3 Histopathological, Biochemical and Safety Evidence

Histopathological and biochemical evidence from a subset of studies (4; 11.8%) confirmed TCA's role in inducing neocollagenesis, with significant increases in type I and III collagen and elastic fibers, especially in studies with higher concentrations [12, 13, 27, 28]. An experimental murine model identified significantly more intense fibroblast activation, greater extracellular matrix deposition, and a more localized and controlled inflammatory response in group treated with focal application of TCA by CROSS when compared to conventional chemical peeling [28].

Safety in high skin phototypes (IV to VI) was addressed in 10 studies (29.4%), confirming efficacy with appropriate protocols and strategies to mitigate post‐inflammatory hyperpigmentation (PIH) [1, 2, 20]. PIH was the most adverse event reported, present in about 20%–25% of studies that detailed complications, especially in studies that employed high concentrations (≥ 65%–100%) and in populations with higher skin phototypes [20]. However, it was described as transient, with complete resolution in a period of 3 to 6 months, particularly when associated with rigorous photoprotection and use of topical depigmenting agents [7, 8, 20].

Other described adverse events were persistent erythema, local edema, and burning sensation, particularly in the first days after the procedure or in protocols that involved subcision [10, 25] and were described as predominantly mild to moderate, self‐limited, with no serious permanent complications [16, 25, 26, 29]. Crust formation was considered an expected finding in CROSS protocols with high concentrations, with average time for spontaneous detachment between 5 and 10 days, without negative impact on final outcomes when post‐procedure guidelines were properly followed [7, 13, 21]. Less frequent adverse events included intense peeling, prolonged pain, and ecchymosis, especially in studies that combined multiple techniques, such as chemical peeling, microneedling, and energy‐based technologies [16, 26, 29]. Still, these events were described as temporary and manageable through conservative measures [16, 25, 26, 29].

## 4 Discussion

The analysis of 34 studies demonstrates that TCA remains a relevant therapeutic pillar in the management of atrophic acne scars, especially through the CROSS technique, which was the most frequently investigated protocol over the last two decades [1, 7, 19, 20, 21, 22, 24]. The focal chemical application of TCA, particularly in ice‐pick scars, promotes localized induction of neocollagenesis and reorganization of the dermal matrix, preserving adjacent skin. This mechanism explains the clinical efficacy achieved with this technique, which in comparative studies has shown outcomes ranging from comparable to inferior relative to ablative technologies such as fractional CO_2_ and Er:Glass lasers, while maintaining lower cost, lower operational complexity, and a broader accessibility profile [7, 22].

The efficacy of TCA is based on well‐characterized biological mechanisms. Experimental and histological evidence demonstrates that its action is not restricted to chemical cauterization but involves activation of reparative biological responses, including intense fibroblast stimulation, increased collagen deposition, and reorganization of extracellular matrix, culminating in consistent dermal remodeling [13, 27, 28]. The increase in type I collagen, along with reorganization and fragmentation of elastic fibers observed after repeated applications of CROSS technique, as well as the significant decrease in serum type III collagen levels following TCA treatment reinforce the biological plausibility of this technique as a focal, selective, and effective intervention in improving skin texture and reducing scar depth [13, 27].

This evidence helps to contextualize the natural and progressive transition observed in literature toward combined protocols in management of atrophic scars, especially in complex presentations, which tend to respond limitedly to isolated therapies [10, 12]. The incorporation of TCA into multimodal strategies, particularly in association with microneedling and subcision, emerges as a consistent trend, especially in treatment of boxcar and rolling scars [5, 9, 10, 12, 25, 30]. Microneedling, when associated with TCA, creates microchannels that facilitate acid penetration and promote a more diffuse inflammatory stimulus, enhancing neocollagenesis [5, 12, 30]. Subcision acts by mechanical release of deep dermal adhesions, allowing TCA to complement tissue remodeling process, evidencing clear synergy between mechanical and chemical stimulant [10, 25]. Protocols that associate TCA with other chemical peels follow a similar rationale, using superficial keratolytic agents as a preparatory peel to reduce stratum corneum resistance and uniformize acid's action [8, 11, 19, 31, 32, 33]. Therefore, while the isolated use of TCA provides clinical benefits, its multimodal combination enhances active delivery and dermal action, thereby yielding superior therapeutic outcomes.

**FIGURE 3:**
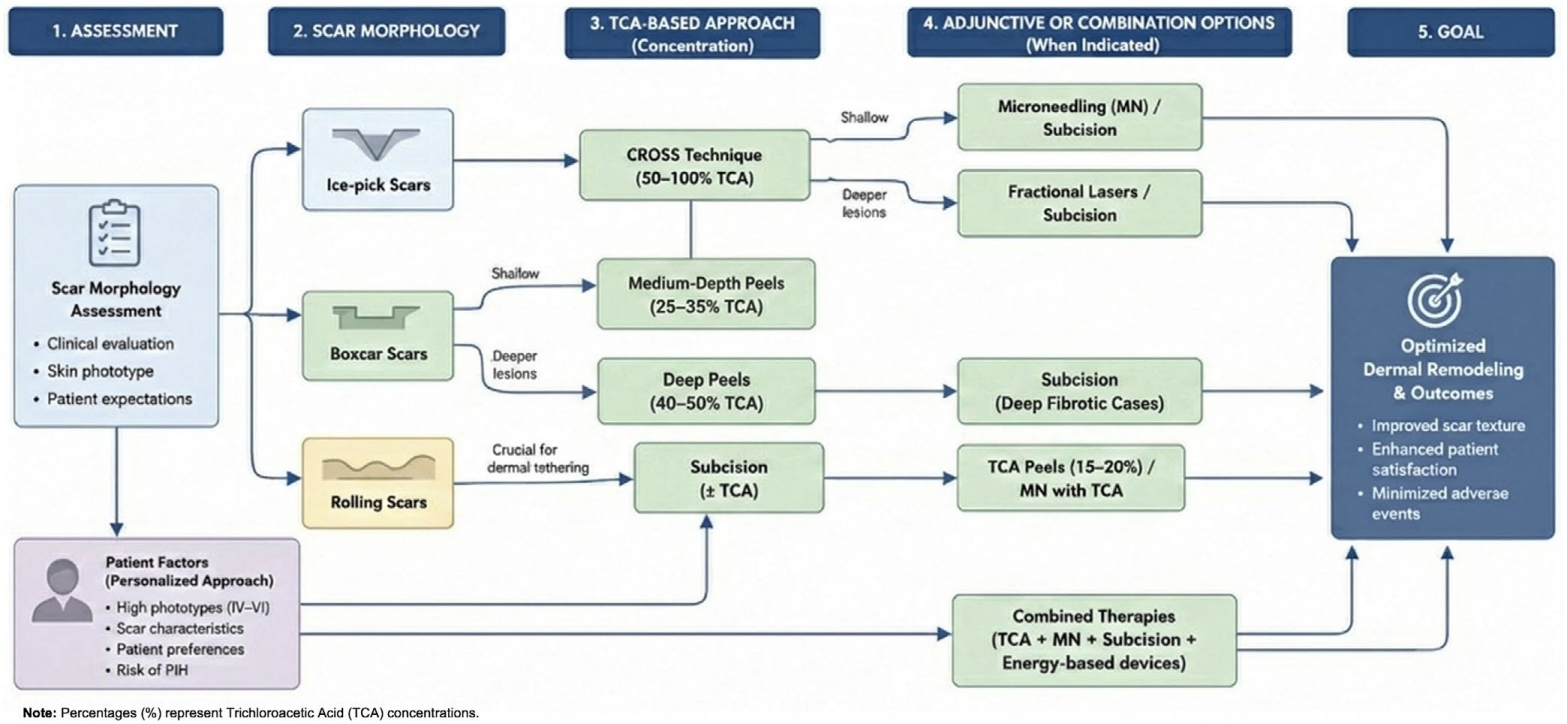
Clinical decision‐making framework for atrophic acne scar treatment with trichloroacetic acid (TCA). The flowchart illustrates a stepwise, personalized approach based on scar morphology (ice‐pick, boxcar, and rolling) and individual patient factors, including Fitzpatrick phototype and risk of post‐inflammatory hyperpigmentation (PIH). It guides the selection of TCA concentrations and application techniques (CROSS technique or chemical peels) as monotherapy or in combination with adjunctive modalities. TCA concentrations indicated (15%–20%, 25%–35%, 40%–50%, and 50%–100%) reflect the protocols most frequently reported in the included studies. CROSS, chemical reconstruction of skin scars; MN, microneedling; PIH, post‐inflammatory hyperpigmentation; TCA, trichloroacetic acid. *Source:* Authors' own illustration.

Therefore, although TCA monotherapy offers significant benefits, multimodal protocols optimize its delivery and intra‐epidermal action, explaining the superior clinical results observed. Based on the synthesized evidence, a practical framework is proposed to guide clinical decision‐making (Figure 3). Most studies highlight that concentration selection is directly associated with penetration depth and patient tolerability, which is further supported by recent evidence reinforcing TCA's favorable safety profile [31, 33, 34]. This reforce the importance of a personalized approach tailored to scar morphology, skin phototype, and individual expectations.

In ice‐pick scars, the CROSS technique (50%–100% TCA) is the gold standard for focal dermal remodeling, with fractional lasers or subcision indicated for resistant cases [7, 19]. Boxcar scars are effectively managed with medium‐depth peels (25%–35% TCA) for shallow variants, while deeper lesions require deep peels (40%–50% TCA) or adjunctive microneedling/subcision to break fibrous septa [5, 10]. Regarding rolling scars, subcision is essential to release dermal anchoring, typically followed by superficial TCA peels (15%–20%) or microneedling with TCA to promote neocollagenesis [25]. For complex or mixed types, multimodal strategies combining TCA with subcision or energy‐based devices outperform monotherapies by addressing multiple pathogenic targets simultaneously [3, 5, 9]. Lastly, for high phototypes (IV‐VI), TCA can be safely used, provided that lower concentrations (e.g., 50%–70% for CROSS, 10%–15% for peels), meticulous skin priming (retinoids, hydroquinone), and strict photoprotection are employed to minimize PIH risk [2, 20].

This scoping review, while comprehensive, is subject to certain limitations. The heterogeneity in study designs, TCA concentrations, application protocols, and outcome measures across the included studies posed challenges for direct comparison or quantitative analysis. The predominance of studies from Asia and the Middle East, while highlighting regional expertise, may limit the generalizability of findings to other populations with different skin types and genetic predispositions. Furthermore, the long publication period (2002–2025) also means that some older studies may not reflect the most current practices or technological advancements. Finally, as a scoping review, the primary aim was to map the existing evidence rather than to critically appraise the quality of individual studies, which is a limitation inherent to this methodology.

In conclusion, TCA remains a fundamental and cost‐effective tool in contemporary clinical practice for the treatment of atrophic acne scars. The evidence suggests a transition from monotherapies to personalized multimodal protocols, where the precision of the CROSS technique and the synergy of combined therapies optimize dermal remodeling while maintaining a high safety profile. The versatility of TCA, whether applied in different concentrations or application techniques, allows for its adaptation to different scar morphologies and skin phototypes, consolidating its role as a versatile agent in clinical practice.

## Author Contributions

Mariana Garcia da Ponte Melo, Joyce Magalhães de Barros: conceptualization, methodology, investigation, formal analysis, and writing, original draft. Ramille Araújo Lima: conceptualization, methodology, writing, review and editing, supervision, and project administration.

## Funding

This study was supported by an internal research fellowship from Christus University. No external funding was received.

## Ethics Statement

The authors have nothing to report.

## Conflicts of Interest

The authors declare no conflicts of interest.

## Data Availability

The data that support the findings of this study are available from the corresponding author upon reasonable request.
